# Is There an Immune Effect of Exercise in Patients with Breast Cancer? A Systematic Review and Meta-Analysis

**DOI:** 10.3390/cancers18040621

**Published:** 2026-02-13

**Authors:** Celia García-Chico, María Merino-País, Simone Lista, Piercarlo Minoretti, Enzo Emanuele, Alejandro Santos-Lozano, Susana López-Ortiz

**Affiliations:** 1 i+HeALTH Strategic Research Group, Department of Health Sciences, Miguel de Cervantes European University, 47012 Valladolid, Spain; cgarciac@uemc.es (C.G.-C.); slista@uemc.es (S.L.); asantos@uemc.es (A.S.-L.); slopezo@uemc.es (S.L.-O.); 2 Studio Minoretti, 23848 Oggiono, LC, Italy; p.minoretti@studiominoretti.it; 3 Department of Social Sciences, Miguel de Cervantes European University, 47012 Valladolid, Spain; 4 2E Science, 27038 Robbio, PV, Italy; enzo.emanuele@2escience.com; 5 Physical Activity and Health Research Group (PaHerg), Research Institute of the Hospital 12 de Octubre (‘imas12’), 28041 Madrid, Spain

**Keywords:** breast neoplasms, resistance training, physical activity, natural killer cells, T-lymphocytes, tumor microenvironment, immunotherapy

## Abstract

**Background/Objectives**: Physical exercise reduces breast cancer (BC) risk and improves survival, yet the biological mechanisms remain incompletely understood. Exercise may modulate systemic immunity and local immune cell infiltration in the tumor microenvironment. In this systematic review and meta-analysis, we examined the effects of exercise on immune cells and immune-related markers in patients with BC. **Methods**: This study followed PRISMA guidelines and was prospectively registered in PROSPERO (CRD420251082444). Four databases (PubMed, Web of Science, Scopus, and Cochrane Library) were searched from inception through December 2025. Randomized controlled trials evaluating exercise interventions in patients with BC or BC survivors and reporting immune cell outcomes were included. Meta-analyses were performed on studies reporting natural killer cells, natural killer cell activity, T-cell subpopulations, and B cells. **Results**: A total of 18 studies involving 911 participants (539 in exercise intervention groups) were included in the systematic review, with eight studies included in meta-analyses. Exercise interventions did not show significant effects on circulating natural killer cell counts, natural killer cell activity, T-cell subpopulations (CD3^+^, CD4^+^, and CD8^+^), or B-cell levels when compared to control groups. **Conclusions**: Exercise does not appear to induce consistent changes in resting circulating immune cell populations in patients with BC or BC survivors, indicating that exercise is immunologically safe while potentially exerting effects beyond circulating cell counts. Further large-scale research is required.

## Simple Summary

Exercise is known to reduce the risk of developing breast cancer and improve survival in patients diagnosed with this disease. However, the biological mechanisms underlying these benefits are not fully understood. One possible explanation is that exercise may enhance the immune system’s ability to fight cancer cells. The immune system plays a crucial role in detecting and eliminating abnormal cells, but cancer cells can sometimes evade this defense. In this systematic review and meta-analysis, we examined all available scientific evidence on how exercise affects immune cells and immune-related markers in breast cancer patients. We also systematically analyzed this evidence to determine whether exercise can improve anti-tumor immunity. Understanding these immune effects may help explain why exercise benefits cancer patients and support the use of exercise programs alongside conventional therapies.

## 1. Introduction

According to the Global Cancer Observatory, breast cancer (BC) is the second most frequently diagnosed cancer worldwide [1], and its incidence is expected to reach 3.2 million cases by 2050 [2]. Despite recent improvements in survival rates, the disease remains a major clinical challenge [3]. BC exhibits considerable biological heterogeneity, including genomic alterations, diverse gene expression patterns, and a complex tumor microenvironment (TME). The TME represents a dynamic cellular ecosystem in which tumor, stromal and immune cells interact [4]. The prognosis of BC is influenced by various cell subpopulations that can either promote or hinder tumor growth [5].

Under physiological conditions, immune cells including cytotoxic T-lymphocytes and natural killer (NK) cells identify and eliminate malignant cells through antigen presentation and cytotoxic activity [6]. Cancer cells can evade immune detection by creating an immunosuppressive microenvironment [7], a key hallmark of cancer [8]. Although BC has been considered immunologically quiescent due to its relatively low somatic mutational burden [9], higher levels of tumor-infiltrating lymphocytes (TILs) in diagnostic biopsies have been associated with improved overall survival in patients with aggressive BC subtypes [5]. These findings highlight the need to develop treatment strategies that improve anti-tumor immune responses.

Immunotherapies, like immune checkpoint inhibitors, block inhibitory pathways that suppress immune system activity [9]. A key objective in immunotherapy is to transform immunologically cold tumors into immune-inflamed hot tumors, thus increasing their responsiveness to various treatments. The infiltration of T and NK cells into the TME and the reduction in tumor hypoxia may facilitate improved immune cell infiltration [10]. However, pharmacological treatments can lead to serious immune-related adverse events, such as pneumonitis, hepatitis, thyroiditis, and skin rash [9], which can negatively impact patients’ outcomes.

Physical activity reduces the risk of BC and improves survival in these patients [11,12], although the specific biological and molecular mechanisms underlying these effects remain poorly understood. Recent evidence suggests that physical exercise may modulate systemic immunity and local infiltration of specific immune cells in the TME [13]. Myokines released during muscle contraction such as interleukin (IL)-6, IL-7, and IL-15 influence the immune system activation, contributing to the systemic effects of physical exercise [13]. Moreover, epinephrine-mediated stimulation of β2-adrenergic receptors on lymphocyte surfaces may induce the mobilization of immune cells into the bloodstream [14,15].

In patients with newly diagnosed BC, a single acute exercise session increased the total number of leukocytes, CD8^+^ T cells, CD19^+^ B cells, NK cells, and CD14^+^CD16^+^ monocytes [16]. This acute exercise-induced NK cell mobilization has also been inversely correlated with tumor size [17]. Moreover, regular exercise may benefit NK cell activity (NKCA) in patients with BC [18], although these results are partly controversial compared to previous research [19,20]. A previous meta-analysis showed that physical exercise, compared to usual care, did not produce statistically significant effects on the number of immune cells (CD8^+^ and CD4^+^ T cells and NK cells) or on NKCA in women with BC [21]. However, given the growing evidence in this field and the increasing mechanistic research on physical exercise and immune system recruitment and activation [14], systematically reviewing the effects of exercise on immune cells associated with cancer may elucidate additional benefits for patients with BC. Therefore, the primary aim of the present systematic review and meta-analysis is to analyze the effects of exercise on immune cells and immune-related markers in patients with BC.

## 2. Materials and Methods

### 2.1. Data Sources and Search Strategy

This systematic review and meta-analysis followed the Preferred Reporting Items for Systematic Reviews and Meta-Analyses (PRISMA) statement [22] (Supplementary Material S1), and the protocol was prospectively registered in the International Prospective Register of Systematic Reviews (PROSPERO) under the registration number CRD420251082444.

To identify eligible studies, a comprehensive literature search was conducted in four electronic databases: PubMed, Web of Science (including Web of Science Core Collection, Current Contents Connect, Derwent Innovations Index, KCI-Korean Journal Database, MEDLINE^®^, ProQuest™ Dissertations & Theses Citation Index, and SciELO Citation Index), Scopus and the Cochrane Library. The following search strategy was used: (“breast cancer” OR “breast neoplasm” OR “mammary cancer” OR “breast tumor” OR “breast tumour”) AND (“exercise” OR “physical activity” OR “strength training” OR “aerobic training” OR “resistance training” OR “endurance training”) AND (“immune” OR “immunity” OR “natural killer” OR “CD8” OR “CD4” OR “lymphocyte” OR “nk cell” OR “neutrophil” OR “monocyte” OR “macrophage” OR “leucocyte”). No language or date filters were applied. The search was conducted from inception through 22 December 2025.

### 2.2. Study Selection

The systematic review included randomized controlled trials (RCTs) and pilot and feasibility studies with a RCT design published in English or Spanish that met the selection criteria based on the Population, Intervention, Comparison, Outcomes and Context (PICOC) framework [23]. Specifically, the criteria included: (i) Population: Patients who were recently diagnosed with BC, undergoing active treatment or BC survivors; (ii) Intervention: Any form of structured physical exercise intervention, defined as physical activity that is planned, structured, repetitive, and purposeful [24]; (iii) Comparison: Usual care control group or other type of exercise intervention; (iv) Outcomes: The primary outcome included immune cells, such as lymphocytes (T and B cells) and NK cells, and secondary outcomes included other molecules and variables related to the immune system; (v) Context: Any form of physical exercise, whether supervised or unsupervised, including home- and center-based programs. Duplicated documents were removed. The search was supplemented by manually reviewing the reference lists of relevant publications to identify additional studies on the topic, reviewing the reference lists of included studies, and searching clinical trial registries in databases (https://clinicaltrials.gov/).

Two independent researchers (C.G.-C. and S.L.-O.) conducted an initial blind screening of the titles and abstracts of the studies to identify those that potentially met the selection criteria. Subsequently, the full text of these articles was reviewed by the same authors to determine their eligibility for final inclusion. Potential disagreements or conflicts were resolved through consensus with a third researcher (M.M.-P.).

The kappa coefficient (κ) and percentage (%) agreement scores were calculated to assess reliability in study selection assessments before consensus. Inter-rater reliability was estimated using κ, with κ > 0.7 indicating a high level of agreement between authors, κ of 0.5–0.7 indicating a moderate level of agreement, and κ < 0.5 indicating a low level of agreement [25].

### 2.3. Data Extraction

From each eligible RCT, two researchers (C.G.-C. and M.M.-P.) extracted the following information and data, if available: main author, year of publication, sample characteristics, intervention type, analyzed outcomes, main results, and baseline and post-intervention results or difference within groups. To ensure the accuracy of the extracted data, a third researcher (S.L.-O.) carefully reviewed and verified the extracted information of each article.

### 2.4. Risk of Bias Assessment

The risk of bias was independently assessed for each RCT by two researchers (C.G.-C. and S.L.-O.) using the Cochrane’s risk of bias 2 (RoB2) [26]. In cases of discrepancies between the scores, a third author (M.M.-P.) was consulted to reach a consensus. The RoB2 tool is structured into a fixed set of domains of bias, focusing on different aspects of trial design, conduct, and reporting. Five domains were assessed: (D1) bias arising from the randomization process; (D2) bias due to deviations from intended interventions; (D3) bias due to missing outcome data; (D4) bias in the measurement of the outcome; and (D5) bias in the selection of the reported results. These categories were classified as having a “high risk,” “low risk” or “some concerns” [26].

### 2.5. Statistical Analysis

The data were analyzed using Review Manager (RevMan) 5.4 software (The Nordic Cochrane Centre, The Cochrane Collaboration, Copenhagen, Denmark). When at least three RCTs analyzed the same outcome, the pooled effect of physical exercise on outcomes related to the immune system in patients who were recently diagnosed with BC, undergoing active treatment or BC survivors was assessed. Since the meta-analysis results combined various types of patients and treatments, we anticipated clinical and methodological heterogeneity between studies and the need for a random-effects model (i.e., DerSimonian and Laird method) to conduct the analyses.

Only studies that specified the mean effect of exercise (baseline and post-intervention data or difference within groups) or provided data that allowed calculation of the mean effect of exercise were included in the meta-analyses. The significance level was established at *p *< 0.05.

The pooled effect estimated from the continuous outcomes of interest was obtained from the mean difference (MD, calculated as post-intervention value − baseline value) and the change standard deviation [SD, calculated using the following formula [27]:

$$\sqrt{{SD}^{2}baseline+{SD}^{2}post\text{-}intervention-(2\times Corr\times SDbaseline\times \;SDpost\text{-}intervention)}$$

To make this calculation, we imputed a correlation value (Corr) of 0.5, which is considered conservative [28]. The pooled result was expressed as the standardized mean difference (SMD) with a corresponding 95% confidence interval (CI).

For the NKCA outcome, we pooled studies that analyzed both cytotoxic and secretory activity of interferon-gamma (IFN-γ) levels. For cytotoxic activity (percentage of cell lysis), when multiple effector-to-target ratios were reported within the same RCT, we selected those with the highest ratio to conduct the meta-analysis.

Statistical heterogeneity was evaluated using a chi-square test (χ^2^), and any inconsistency was quantified using the I^2^ statistic. These values were interpreted as follows: 0–40% indicated low heterogeneity, 30–60% indicated moderate heterogeneity, 50–90% indicated substantial heterogeneity, and values greater than 75% were considered indicative of considerable heterogeneity [29].

## 3. Results

**Figure 1:**
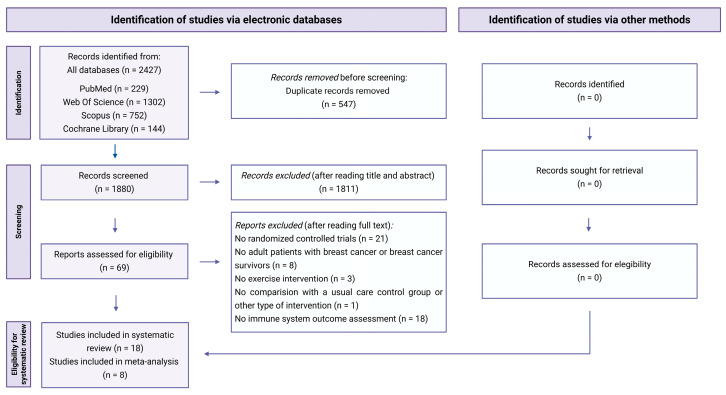
Flowchart of studies included in systematic review and meta-analysis.

Initially, a total of 2427 records were identified for screening from the four electronic databases. After removing 547 duplicates, 1880 documents were screened (Supplementary Materials S2). Finally, 69 full-text articles were reviewed and 18 were selected for inclusion in the systematic review, of which eight were included in the meta-analysis (Figure 1). The inter-rater level of agreement was classified as high in the study selection process before consensus (κ = 0.89; 95%CI = 0.76 to 1.02).

### 3.1. Characteristics of Included Studies

Table 1 provides an overview of the specific sample characteristics of the RCTs [30,31,32,33,34,35,36,37,38,39,40,41,42,43,44,45,46,47] included in the systematic review. Among all the RCTs, three were conducted in the United States [30,36,43], three in Australia [32,33,39], one divided into two studies in Sweden [38,41], two in Spain [34,40], and one in Canada [31], Germany [35], Egypt [37], Korea [42], the United Kingdom [44], Denmark [45], The Netherlands [46] and China [47].

The total sample size comprised 911 analyzed participants, with 539 allocated to an intervention group. The sample size of the individual RCTs included ranged from 16 [30] to 240 [38,41] participants. In 16 of the 18 included studies, all the participants were women, while in the remaining two studies, sex was not specified [46,47]. Regarding BC stage, the RCTs were conducted with participants with stage I–III [36,43,44,46], I–IIIA [31,32,33,38,41], stage I [37], and I–II [34] BC. Another RCT was conducted with women with primary moderate- or high-risk BC [35], while five RCTs did not specify the BC stage in their selection criteria [30,39,40,45,47].

The exercise interventions were conducted in patients who were planning to undergo primary breast surgery [36]; had undergone modified radical mastectomy (or unilateral axillary lymph node dissection) [47]; had completed surgery, radiotherapy, and/or chemotherapy [32,33], with or without current tamoxifen or anastrozole therapy [31]; were scheduled for chemotherapy [46] or in the process of receiving it [34,37]; were planning to complete adjuvant [35] or neoadjuvant [35,45,46] chemotherapy or a further chemotherapy regimen or were undergoing it [38,41]; had completed cancer treatment at least six months before [43] or within the previous two years [39]; had completed chemotherapy or radiotherapy five years ago or less [40]; were treated with surgery, chemotherapy and/or radiotherapy that was completed more than two years ago [42] or within the previous four years [30]; or received their last treatment at least two months before (no longer than five years prior) [44].

Table 2 summarizes the characteristics of the interventions and outcomes described and analyzed in the systematic review. The types of exercise analyzed included aerobic training (AT) [31,35,37,39,44], resistance training (RT) [32,33,35,42], combined AT + RT [30,34,36,40,45,46,47], and high-intensity interval training (HIIT) combined with both AT and RT [38,41,43]. Regarding exercise interventions, each protocol consisted of two [35,36,40,42,43,45,46,47] or three [30,32,33,34,37,38,39,41,42,43,44,45,47] sessions per week, with a total intervention duration ranging from 29.3 days [36] to 52 weeks [43], although most interventions lasted 12 [35,39,42,47] or 16 weeks [32,33,38,40,41].

AT [31,35,37,38,39,44] was performed for 15 [31,37] to 60 min [30,38,41] per session and intensity was quantified using the rate of perceived exertion (RPE) scale [35,38,41], peak oxygen consumption (70–75% VO_2peak_) [31], maximum oxygen consumption (55–80% VO_2max_) [37,44], and maximum power (50–65% work rate) [39].

For RT, intensity was determined by the weight lifted, based on the RPE scale, or by the percentage of one-repetition maximum (1RM), which ranged from 40% [42] to 80% 1RM [32,33,42]. The number of prescribed exercises ranged from three [45] to 10 [35,40], with seven exercises being the most common [30,32,33,47]. The training volume included a variety regarding the number of sets and repetitions, with two or three sets and 8–12 repetitions representing the most frequently used.

In studies combining AT and RT, AT was performed for 20 [34,38,41] to 30–45 [36] minutes per session, and intensity was quantified using maximum heart rate (75 to ≥85% HRmax) [30,45], peak oxygen consumption (60–70% VO_2peak_) [34], or an RPE scale (6 to 7–8 on a 0–10 point scale and 12–13 to 16–18 on a 6–20 point scale) [40,45,47], as well as maximum power (50–80% work rate) [46]. RT intensity was determined by the percentage of 1RM (40 to even >80% 1RM) [38,41,46,47] or by an RPE scale (6–7 on a 0–10 point scale and 13 to 15 on a 6–20-point scale) [40,47]. Moreover, two RCTs combined RT with HIIT [38,41,45], with intensity prescribed using an RPE scale (>16 on a 6–20-point scale) [38,41,45] or based on maximum heart rate (≥85% HRmax) [45].

### 3.2. Risk of Bias Assessment Results

Most of the studies included in the systematic review had some concerns related to their risk of bias due to unreported information. In these studies, the domains with a higher lack of information were D1, D2 and D3. Two studies [31,43] had a low risk of bias while one [30] showed a high risk due to concerns in domains D2, D3 and D4. A detailed description of the risk of bias assessment using the RoB2 algorithm for individual domains is included in Supplementary Material S3.

### 3.3. Synthesis

We meta-analyzed a total of eight studies [30,31,32,34,35,37,42,46] that assessed six outcomes.

#### 3.3.1. Natural Killer Cells and Natural Killer Cell Activity

**Figure 2:**
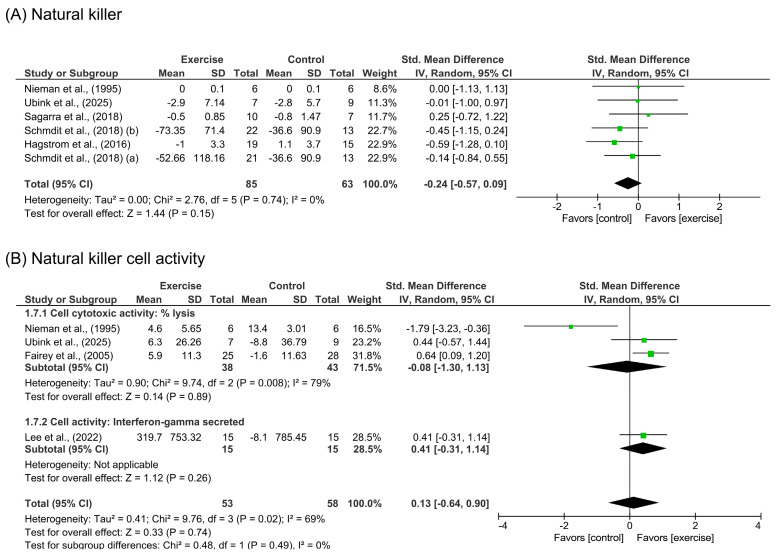
Meta-analysis results for (**A**) natural killer cells [30,32,34,35,46] and (**B**) natural killer cell activity [30,31,42,46].

Five studies [30,32,34,35,46] comprising six intervention arms (*n* = 148 participants) evaluated the effects of exercise on circulating NK cell counts. The pooled analysis did not show significant benefits [SMD = −0.24 (95%CI: −0.57 to 0.09), *p* = 0.15] in those who exercised compared to a control group, as shown in Figure 2A. We did not find heterogeneity in these analysis studies (I^2^ = 0%; *p* = 0.74). A pooled sensitivity sub-analysis of three studies [30,34,46] was performed to study the effects of combined exercise (AT + RT), showing similar results [SMD = 0.09 (95%CI: −0.50 to 0.68), *p* = 0.77]. The pooled sensitivity analysis for long-term (≥8 weeks) exercise interventions, performed by removing the study of Ubink et al. [46], also showed no significant effects [SMD = −0.27 (95%CI: −0.62 to 0.08), *p* = 0.13].

A total of four studies assessed NKCA through NK cell cytotoxic activity [30,31,46] and secretory activity of IFN-γ levels [42]. The pooled result of physical exercise did not show a significant effect on this outcome [SMD = 0.13 (95%CI: −0.64 to 0.90), *p* = 0.74] with substantial heterogeneity (I^2^ = 69%; *p* = 0.02), as represented in Figure 2B. We also conducted a sensitivity sub-analysis to assess the isolated effects on cytotoxic activity [30,31,46]. However, the results remained non-significant [SMD = −0.08 (95%CI: −1.30 to 1.13), *p* = 0.89].

#### 3.3.2. T Cells

**Figure 3:**
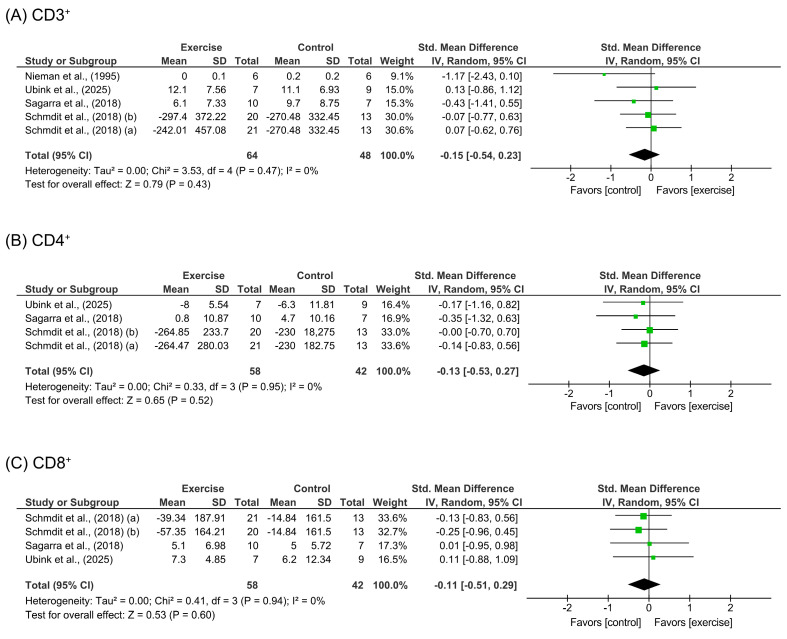
Meta-analysis results for (**A**) CD3^+^ [30,34,35,46]; (**B**) CD4^+^ [34,35,46]; (**C**) CD8^+^ [34,35,46].

Physical exercise interventions did not demonstrate significant effects on any T-cell subpopulation, as shown in Figure 3. For total lymphocytes (CD3^+^), pooled data from 112 participants [30,34,35,46] showed no differences between intervention and control groups [SMD = −0.15 (95%CI: −0.54 to 0.23), *p* = 0.43]. Similarly, no significant improvements were observed for CD4^+^ [SMD = −0.13 (95%CI: −0.53 to 0.27), *p* = 0.52; *n* = 100 participants] [30,34,35,46] and CD8^+^ subpopulations [SMD = −0.11 (95%CI: −0.51 to 0.29), *p* = 0.60; *n* = 100 participants] [34,35,46].

A sensitivity sub-analysis pooling the effects of three studies [30,34,46] performing combined training interventions showed no significant differences for CD3^+^ [SMD = −0.41 (95%CI: −1.09 to 0.28), *p* = 0.25; *n* = 45 participants]. Moreover, a sensitivity sub-analysis of long-term exercise interventions, performed by removing the study of Ubink et al. [46], showed similar effects [SMD = −0.21 (95%CI: −0.64 to 0.22), *p* = 0.33; *n* = 96 participants]. All sensitivity analysis results are shown in Supplementary Material S4.

#### 3.3.3. B Cells

**Figure 4:**
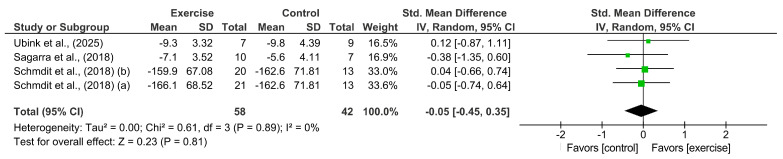
Meta-analysis results for B cells [34,35,46].

The pooled analysis of four arms from three studies [34,35,46] (see Figure 4) revealed no significant differences between participants allocated to an intervention group and those in the control group [SMD = −0.05 (95%CI: −0.45 to 0.35), *p* = 0.81; 100 participants].

## 4. Discussion

The findings of this systematic review and meta-analysis of RCTs demonstrate that physical exercise interventions, regardless of type, do not appear to modify the levels of immune cells (CD3^+^, CD4^+^, CD8^+^, and NK) or immune-related variables (NKCA) in patients with BC or BC survivors. These results also indicate that physical exercise did not negatively affect the immune system in this population.

Exercise guidelines for patients with cancer recommend achieving at least 150 min of moderate-intensity physical activity and performing RT twice a week [48]. However, patients with cancer tend to progressively decrease their levels of physical activity from diagnosis through treatment and follow-up [49], which may negatively impact disease prognosis and survival [50]. Despite strong epidemiological evidence supporting the anticancer effects of exercise [51], the biological mechanisms underlying these benefits remain incompletely understood and are recognized as a main research priority [48,52]. Among these molecular effects, stimulating the immune system can be an effective way to prevent the risk and progression of primary tumors [53].

Exercise induces a biphasic response, whereby circulating leukocytes initially increase in the bloodstream and then decrease below resting levels within hours following the exercise session [14,54]. This post-exercise lymphopenia is typically observed among natural killer cells and CD8^+^ T cells. However, rather than suppressing immune competency, this transient state may even enhance immune surveillance and regulation [55]. In fact, a single bout of moderate-intensity aerobic exercise has been shown to induce an immediate increase of T cells, followed by a subsequent reduction in the proportion of leukocytes in patients with cancer [16,56]. The repeated and transient immunosurveillance status could contribute to decreased systemic inflammation and illness incidence [57]. In line with this process, immune cell function may also be slightly altered by exercise through changes in cytokine production and cytotoxic activity. Moreover, exercise can alter circulating immune cells by promoting their redistribution to other tissues and organs [54], which could affect intra-tumor infiltration of cytotoxic T cells [57]. Despite these proposed mechanisms, our pooled results did not reveal a statistically significant change in the number of immune cells or NKCA in patients with BC or BC survivors.

The results regarding the effects of exercise on NK cells and NKCA are consistent with those of previous meta-analyses conducted in cancer survivors [20,58]. Although high-intensity interval training may also enhance NKCA, moderate-intensity exercise appears to primarily promote immune surveillance and cell functions. Even short bouts of moderate-intensity AT can induce NK cell mobilization into the bloodstream [59]. Therefore, although the pooled effect from our meta-analysis did not show significant longitudinal changes in NK cell outcomes after exercise interventions, acute exercise-induced immune responses may still be biologically relevant and contribute to the overall anticancer effects of exercise.

Moderate-intensity exercise may also modify B- and T-cell distribution and activity [59], although the specific effects on B cell-related outcomes remain unclear [60]. Regarding T cells, a meta-analysis conducted on individuals with non-communicable diseases showed that AT significantly improved the counts of CD8^+^ and CD4^+^ T cell compared to a non-exercised control group, suggesting a potential long-term effect of exercise [61]. Another study reported that physical activity interventions significantly increased CD4^+^ cell counts in adults [62]. However, our results did not show statistically significant findings for these outcomes, which could be due to characteristics and alterations of the immune system specific to patients with BC and BC survivors.

The human immune system varies considerably among individuals but remains relatively stable over time within each person [63]. However, people with cancer may experience multiple immunological alterations during treatment, including immunosuppression and cancer-related systemic inflammation. Chemotherapy regimens exert cytotoxic effects that can affect various immune cell subsets, influencing the distribution and composition of circulating lymphocytes and TILs [64]. Therefore, the number of immune cells generally decreases throughout chemoradiotherapy in these patients [65]. The reduction in absolute lymphocyte count may persist up to 12 months after completing chemotherapy [66]. Radiotherapy can also stimulate or suppress immune responses, either promoting anti-tumor immunity by activating cytotoxic T cells through damage-associated molecular patterns or facilitating tumor progression through immunosuppressive mechanisms [67]. Moreover, BC surgery also induces a pro-inflammatory response and leukocytosis with decreased NKCA [68]. In summary, variation in immune system cell count and activity during cancer progression may affect the detection of changes after long-term exercise programs, especially in patients undergoing treatment. Further research is needed to understand the effects of physical exercise at different stages of cancer progression, while accounting for variations in immune cell count and activity throughout the process.

Exercise type, intensity, and duration can also affect the results obtained. Acute bouts of exercise have been shown to induce lymphocytosis, with both CD4^+^ and CD8^+^ T cells increasing in an intensity-dependent manner. Additionally, rest duration between bouts of exercise could differentially modulate T-cell responses, as short recovery periods followed by subsequent exercise can enhance CD8^+^ T-cell mobilization [69]. Therefore, while moderate-intensity exercise appears to promote immune surveillance and reduce systemic chronic inflammation, HIIT may induce acute immune activation. However, the sustained effects of this type of training remain poorly understood [59]. Previous research also suggests that strenuous and prolonged exercise could even decrease the activity and number of innate immune system cells [70]. Therefore, further studies investigating and comparing different types and methodologies of exercise training could provide valuable insights into the immunological and clinical adaptations for patients with BC.

The TME also promotes immunosuppressive mechanisms that can impair anti-tumor immunity. Inflammation, as a central mediator of immune function, can be modulated by physical exercise [71]. Several studies have demonstrated potential effects of exercise, especially combined training, on pro-inflammatory markers such as IL-6, IL-8, and tumor necrosis factor-alpha [72,73]. Although specific effects on immune cells and markers remain limited, findings on inflammatory molecules support the potential benefits of physical exercise and could serve as prognostic indicators in patients with BC, given their relationship with the immune system.

Several limitations of the present systematic review and meta-analysis must be acknowledged to interpret the results. We observed heterogeneity in participant characteristics both between and within studies (e.g., disease stage and type of treatment), which might have potentially confounded the effects of exercise interventions. In fact, some studies did not specify the details of BC stage, which limits the generalizability of the results. Moreover, the meta-analyses pooled the results of different types of exercise interventions, such as AT, RT and combined training, which limits the possibility to detect specific exercise-induced effects. Methodological variation was also observed among the different pooled interventions in terms of type, intensity, volume and total study duration. Although previous evidence has identified high-intensity aerobic and RT as the most promising types of exercise for reducing inflammation [74], moderate-intensity AT seems to promote immune regulation [59]. Exercise adherence and compliance could also affect the observed results obtained in the individual RCTs. Therefore, future research should also consider discrepancies in the molecular effects induced by exercise depending on its characteristics. In addition, immune markers were measured before and after a period of exercise intervention, which limits the ability to obtain results regarding the acute response to exercise. Regarding between-study variability, substantial heterogeneity was observed in certain sensitivity subgroup analyses, particularly for NK cell counts in long-term exercise interventions and for NK cell cytotoxic activity. Although no considerable heterogeneity (I^2^ > 75%) was detected in the overall meta-analyses for any of the immune outcomes examined, this heterogeneity may be influenced by specific intervention characteristics or by cytotoxic activity assessment techniques. Publication bias could not be assessed in the meta-analyses due to the small number of RCTs, as *p*-value-based tests may underestimate their presence when few studies are available [75]. Finally, most RCTs measured circulating immune markers. Future research should focus on analyzing the impact of exercise interventions on TILs to broaden knowledge of the potential immune effects within tumor tissue. Despite these limitations, our study provides novel insights into the immune-mediated effects induced by physical exercise interventions in patients with BC and BC survivors.

## 5. Conclusions

Physical exercise does not appear to induce significant changes in resting circulating immune cell populations in patients with BC or BC survivors across different types of exercise, suggesting a neutral effect on baseline immune status in this population. Although current evidence provides a molecular rationale for exercise-induced immune modulation, the limited number of studies with robust methodologies and adequate sample sizes limits the translation of these findings into practical recommendations for both AT and RT. Therefore, further well-designed and larger-scale studies are warranted to elucidate the effects of exercise on the immune system in patients with BC, while accounting for disease stage, treatment status, and the potential impact of systemic and local therapies.

## Figures and Tables

**Table 1:** Characteristics of the participants included in the systematic review.

First Author (Year)	Age (Mean ± SD)	% Women Sample Size (Randomized)	Sample Size (Analyzed)	Treatment Type and Timing or Clinical Situation	Stage of Cancer
Nieman et al., (1995) [30]	**EG:** 60.8 ± 4.0 **CG:** 51.2 ± 4.7	100% **EG:** *n* = 8 **CG:** *n* = 8	**EG:** *n* = 6 **CG:** *n* = 6	Surgery, chemotherapy and/or radiotherapy within the previous four years	Not specified
Fairey et al., (2005) [31]	**EG:** 59.0 ± 5.0 **CG:** 58.0 ± 6.0	100% **EG:** *n* = 25 **CG:** *n* = 28	**EG:** *n* = 25 **CG:** *n* = 28	Completed surgery, radiotherapy, and/or chemotherapy with or without current tamoxifen or anastrozole therapy use	Stage I–IIIA
Hagstrom et al., (2016) [32] (*ANZCTR #12612000346875 study*)	**EG:** 51.2 ± 8.5 **CG:** 52.7 ± 9.4	100% **EG:** *n* = 20 **CG:** *n* = 19	**EG:** *n* = 19 **CG:** *n* = 15	Completed surgery, radiotherapy, and/or chemotherapy	Stage I–IIIA with no evidence of recurrent disease
Hagstrom et al., (2018) [33] (*ANZCTR #12612000346875 study*)	**EG:** 50.8 ± 8.3 **CG:** 52.1 ± 8.5	100% **EG:** *n* = 19 **CG:** *n* = 14	**EG:** *n* = 19 **CG:** *n* = 14	Completed surgery, radiotherapy, and/or chemotherapy	Stage I–IIIA
Sagarra et al., (2018) [34]	**EG:** 50.0 ± 5.5 **CG:** 53.1 ± 6.8	100% **EG:** *n* = 11 **CG:** *n* = 11	**EG:** *n* = 10 **CG:** *n* = 7	During chemotherapy	Stage I–II
Schmidt et al., (2018) [35]	**EG1** (RT): 53.0 ± 12.6 **EG2** (AT): 56.0 ± 10.2 **CG:** 54.0 ± 11.2	100% **EG1**: *n* = 24 **EG2**: *n* = 29 **CG:** *n* = 28	**EG1**: *n* = 21 **EG2**: *n* = 20 **CG:** *n* = 26	Planned adjuvant or neoadjuvant chemotherapy or further chemotherapy regimen	Primary moderate- or high-risk BC
Ligibel et al., (2019) [36]	**EG:** 52.3 ± 9.6 **CG:** 53.1 ± 7.9	100% **EG:** *n* = 26 **CG:** *n* = 22	**EG:** *n* = 14 **CG:** *n* = 11	Planning to undergo primary breast surgery	Stage I–III
Ashem et al., (2020) [37]	**EG:** 45.0 ± 3.3 **CG:** 45.1 ± 3.0	100% **EG:** *n* = 15 **CG:** *n* = 15	**EG:** *n* = 15 **CG:** *n* = 15	Undergoing chemotherapy	Stage I
Mijwel et al., (2020) [38] (*OptiTrain Breast Cancer Trial*)	**EG1** (RT): 52.7 ± 10.3 **EG2** (AT): 54.4 ± 10.3 **CG:** 52.6 ± 10.2	100% **EG1**: *n* = 79 **EG2**: *n* = 80 **CG:** *n* = 81	**EG1**: *n* = 65 **EG2**: *n* = 60 **CG:** *n* = 57	Undergoing adjuvant chemotherapy (consisting of anthracyclines, taxanes, or a combination of both)	Stage I–IIIA
Toohey et al., (2020) [39]	**EG1** (MIAT): 65.0 ± 7.7 **EG2** (HIIT): 60.0 ± 8.1 **CG:** 61.0 ± 7.9	100% **EG1**: *n* = 5 **EG2**: *n* = 6 **CG:** *n* = 6	**EG1**: *n* = 5 **EG2**: *n* = 6 **CG:** *n* = 6	Completed cancer treatment within the previous two years	Not specified
Pagola et al., (2020) [40]	**EG1** (RT + HIAT): 47.0 ± 7.0 **EG2** (RT + MIAT): 51.0 ± 6.0	100% **EG1**: *n* = 13 **EG2**: *n* = 10	**EG1**: *n* = 13 **EG2**: *n* = 10	Chemotherapy or radiotherapy completed five years ago or less	Not specified
Hiensch et al., (2021) [41] (*OptiTrain Breast Cancer Trial*)	**EG1** (RT): 52.2 ± 10.1 **EG2** (AT): 53.9 ± 7.4 **CG:** 52.9 ± 10.1	100% **EG1**: *n* = 79 **EG2**: *n* = 80 **CG:** *n* = 81	**EG1**: *n* = 30 **EG2**: *n* = 27 **CG:** *n* = 29	Undergoing adjuvant chemotherapy	Stage I–IIIA
Lee et al., (2022) [42]	**EG:** 54.7 ± 5.1 **CG:** 55.4 ± 4.3	100% **EG:** *n* = 15 **CG:** *n* = 15	**EG:** *n* = 15 **CG:** *n* = 15	Surgery, chemotherapy, or radiotherapy completed more than two years ago	Stage I–IIIA
Brown et al., (2023) [43]	**EG:** 59.2 ± 8.1 **CG:** 58.9 ± 8.4	100% **EG:** *n* = 87 **CG:** *n* = 90	**EG:** *n* = 86 **CG:** *n* = 88	Cancer-directed therapy completed ≥ 6 months before	Stage I–III
Arana Echarri et al., (2023) [44]	**Total:** 56.0 ± 6.0	100% **EG1**: *n* = 10 **EG2**: *n* = 10	**EG1**: *n* = 10 **EG2**: *n* = 10	Last treatment received at least two months before (no longer than five years prior)	Stage I–III
Kjeldsted et al., (2025) [45]	Not specified (≥18 years of age)	100% **EG:** *n* = 50 **CG:** *n* = 52	**EG:** *n* = 49 **CG:** *n* = 52	Planning to undergo neoadjuvant chemotherapy	Not specified
Ubink et al., (2025) [46]	**EG:** Median = 53.0; interquartile range = 16 **CG:** Median = 44.0; interquartile range = 26	Sex not specified **EG:** *n* = 11 **CG:** *n* = 9	**EG:** *n* = 7 **CG:** *n* = 9	Scheduled for neoadjuvant chemotherapy with four cycles of two- or three-weekly Adriamycin and cyclophosphamide, followed by weekly paclitaxel	Stage I–III
Yijing et al., (2025) [47]	**EG1** (LIRT): 49.1 ± 9.4 **EG2** (MIRT): 46.7 ± 10.98 **CG:** 51.5 ± 4.6	Sex not specified **EG1**: *n* = 38 **EG2**: *n* = 38 **CG:** *n* = 38	**EG1**: *n* = 36 **EG2**: *n* = 37 **CG:** *n* = 37	Had undergone modified radical mastectomy (or unilateral axillary lymph node dissection)	Not specified

Abbreviations: AT, aerobic training; BC, breast cancer; CG, control group; EG, experimental group; EG1, experimental group-1; EG2; experimental group-2; HIIT, high-intensity interval training; HIAT, high-intensity aerobic training; LIRT, low-intensity resistance training; MIRT, moderate-to-high-intensity resistance training; MIAT, moderate-intensity aerobic training; RT; resistance training.

**Table 2:** Characteristics of the interventions and outcomes described and analyzed in the systematic review.

Author (Year)	Intervention		Outcome	Main Results
Nieman et al., (1995) [30]	**EG**	**Type of exercise:** Supervised AT and RT **Intensity:** 75% HRmax (AT); weight progressively increased (RT) **Volume:** 3 sessions per week; 60 min each session: 30 min (AT) + 2 sets of 12 repetitions of 7 exercises (RT) **Total duration:** 8 weeks	CBC (*automated hematology analyzer*, *Coulter*) Percentage of NK (CD3^−^CD16^+^CD56^+^) and T (CD3^+^)-cell subsets (*lymphocyte phenotyping*) NKCA (*chromium release analysis*)	No significant differences between groups in CBC, percentage of immune cells, and NKCA
	**CG**	**Type of intervention:** Usual care **Total duration:** 8 weeks		
Fairey et al., (2005) [31]	**EG**	**Type of exercise:** Supervised AT **Intensity:** 70–75% VO_2peak_ **Volume:** 3 sessions per week of 15 min (weeks 1–3); 20 min (weeks 4–6); 25 min (weeks 7–9); 30 min (weeks 10–12); and 35 min (weeks 13–15) **Total duration:** 15 weeks	Standard hematological variables (*automated hematology analyzer*, *Coulter*) Neutrophil size, granularity, and oxidative burst (*flow cytometry*) NKCA (*chromium-51 release assay*) Blood mononuclear cell phenotypes (*immunofluorescence assay by flow cytometry*) Mononuclear cell proliferative capacity: unstimulated and PHA-stimulated ([^3^*H*]*thymidine uptake assay*)	↑ NKCA in the EG ↑ Mononuclear cell proliferative capacity (unstimulated) in the EG No significant differences between groups in standard hematological variables; neutrophil size, granularity, and oxidative burst; blood mononuclear cell phenotypes; and mononuclear cell proliferative capacity (PHA-stimulated)
	**CG**	**Type of intervention:** No exercise and were asked not to begin a structured exercise program **Total duration:** 15 weeks		
Hagstrom et al., (2016) [32] (*ANZCTR #12612000346875 study*)	**EG**	**Type of exercise:** Supervised RT **Intensity:** 8RM (80% 1RM) **Volume:** 3 sessions per week; 3 sets of 8–10 repetitions of 6–7 exercises **Total duration:** 16 weeks	NK and NKT cell count (*flow cytometry* and *FACS*) Functional markers on NK and NKT cells: granzyme B and perforin (*flow cytometry* and *FACS*) IFN-γ (*PMA*/*Ionomycin*/*Brefeldin A stimulation*, *flow cytometry*, and *FACS*) CBC (*automated hematology analyzer*, *Coulter STKS*)	No significant differences between groups in NK and NKT cell counts; functional markers on NK and NKT cells; IFN-γ; and CBC
	**CG**	**Type of intervention:** No exercise **Total duration:** 16 weeks		
Hagstrom et al., 2018 [33] (*ANZCTR #12612000346875 study*)	**EG**	**Type of exercise:** Supervised RT **Intensity:** 8RM (80% 1RM) **Volume:** 3 sessions per week; 3 sets of 8–10 repetitions of 6–7 exercises **Total duration:** 16 weeks	Leucocyte telomere length (*qPCR*)	No significant differences between groups in leucocyte telomere length
	**CG**	**Type of intervention:** No exercise **Total duration:** 16 weeks		
Sagarra et al., (2018) [34]	**EG**	**Type of exercise:** Supervised AT + RT **Intensity:** 60–70% (AT) + weights of 0.5–1 kg on the non-intervened side (RT) + two psychosocial support sessions **Volume:** 3 sessions per week; 10 min warm-up + 20 min (AT) + 15 min (RT) + 5 min cool down **Total duration:** 18–22 weeks	T cells (CD3^+^, CD4^+^, CD8^+^, CD4^+^/CD8^+^ ratio) (*not specified*) NK cells (*not specified*) B cells (*not specified*) Serum Igs G, M, E and A (*not specified*)	↓ B cells in the EG ↓ Igs G, M, E and A in the EG ↓ IgG in the CG ↑ CD8^+^ T cells in both groups ↑ CD3^+^ and CD4+ in the CG
	**CG**	**Type of intervention:** Two psychosocial support sessions **Total duration:** 16 weeks		
Schmidt et al., (2018) [35]	**EG1**	**Type of exercise:** Supervised RT **Intensity:** Hypothetical 50% of the maximum weight and progression based on the Borg scale **Volume:** 2 sessions per week; one set of 20 repetitions of 10 exercises **Total duration:** 12 weeks	T cells (CD3^+^, CD4^+^ and CD8^+^) (*flow cytometry* and *FACS*) NK cells (*flow cytometry* and *FACS*) B cells (*flow cytometry* and *FACS*) V **γδ** T cells (*flow cytometry* and *FACS*)	↓ CD4^+^ T cells in all groups ↓ B cells in all groups ↓ CD8^+^ T cells in EG2 ↓ NK cells in EG2 No significant differences between groups in CD4^+^ and CD8^+^ T cells in EG1 and CG; NK cells in EG1 and CG; and γδ T cells
	**EG2**	**Type of exercise:** Supervised AT **Intensity:** RPE 11–14 **Volume:** 2 sessions per week; 45 min for each session (10 min warm-up; 25–30 min exercise; and 5 min cool down) **Total duration:** 12 weeks		
	**CG**	**Type of intervention:** Usual care **Total duration:** 12 weeks		
Ligibel et al., (2019) [36]	**EG**	**Type of exercise:** Supervised RT and AT + additional unsupervised AT **Intensity:** Moderate **Volume:** 2 sessions per week; 30–45 min (AT) + 20 min (RT) of six exercises **Total duration:** mean 29.3 days	T cells (CD4^+^ and CD8^+^) (*fluorescence IHC* and *multiplexed immunofluorescence*) FOXP3^+^ regulatory T cells (*fluorescence IHC* and *multiplexed immunofluorescence*) NK cells (CD56^+^) (*fluorescence IHC* and *multiplexed immunofluorescence*) Macrophages (CD163^+^) (*fluorescence IHC* and *multiplexed immunofluorescence*)	No significant differences between groups in CD4^+^ and CD8^+^ T cells; NK cells (CD56^+^); macrophages (CD163^+^) and FOXP3^+^
	**CG**	**Type of intervention:** Mind–body control (surgical preparation program consisting of a book and a relaxation audio guide) **Total duration:** mean 29.3 days		
Ashem et al., (2020) [37]	**EG**	**Type of exercise:** Supervised AT **Intensity:** 60–80% VO_2max_ (progressively increased) **Volume:** 3 sessions per week for 15 min (weeks 0–2); 20 min (weeks 3–5); 25 min (weeks 6–8); 30 min (weeks 9–11); 35 min (weeks 12–14); 40 min (weeks 15–18); and 45 min (>18 weeks) **Total duration:** 20 weeks	IgA (*phlebotomy*)	↑ IgA in EG ↑ IgA in EG vs. CG
	**CG**	**Type of intervention:** No intervention **Total duration:** 20 weeks		
Mijwel et al., (2020) [38] (*OptiTrain Breast Cancer Trial*)	**EG1**	**Type of exercise:** Supervised RT-HIIT **Intensity:** 70–80% 1RM (progression when more than 12 repetitions could be performed) (RT) + 16–18 RPE (HIIT) **Volume:** 3 sessions per week; 60 min for each session: 2–3 sets of 8–12 repetitions of 8 exercises (RT) + 3 × 3 min bouts (HIIT) **Total duration:** 16 weeks	Circulating blood cell concentrations (neutrophils and lymphocytes) (*clinical laboratory analysis*)	No significant differences between groups in circulating blood cell concentrations
	**EG2**	**Type of exercise:** Supervised AT-HIIT **Intensity:** RPE 13–15 (AT) + RPE 16–18 (HIIT) **Volume:** 3 sessions per week; 60 min for each session: 20 min AT + 3 × 3 min bouts (HIIT) **Total duration:** 16 weeks		
	**CG**	**Type of intervention:** Usual care (information about physical activity) **Total duration:** 16 weeks		
Toohey et al., (2020) [39]	**EG1**	**Type of exercise:** Supervised continuous MIAT **Intensity:** 55–65% Wmax (progressively increased); RPE 9–13 **Volume:** 3 sessions per week; 5 min warm-up + 20 min MIAT + 5 min cool down **Total duration:** 12 weeks	Saliva IgA (*IPRO Fluid Collection kit*)	No significant differences between groups in IgA
	**EG2**	**Type of exercise:** Supervised HIIT **Intensity:** As hard as possible (cadence 95–115 RPM to ensure consistency) **Volume:** 3 sessions per week; 5 min warm-up + 4–7 × 30 s bouts with 2 min of active recovery between bouts (HIIT) + 5 min cool down **Total duration:** 12 weeks		
	**CG**	**Type of intervention:** Usual care **Total duration:** 12 weeks		
Pagola et al., (2020) [40]	**EG1**	**Type of exercise:** Supervised RT + HIAT **Intensity:** RPE 7–8 (HIAT) + RPE 6–7 (RT) **Volume:** 2 sessions per week; 75 min for each session: 10 min warm-up + 35 min (HIAT) + 30–35 min RT (2–3 sets of 8–12 reps of 8–10 exercises) **Total duration:** 16 weeks	Concentration of neutrophils and lymphocytes (*automated hematology analyzer*) NLR (*neutrophils*/*lymphocytes*)	No significant differences in NLR
	**EG2**	**Type of exercise:** Supervised RT + unsupervised MIAT **Intensity:** RPE 6 (MIAT) + RPE 6–7 (RT) **Volume:** >150 min/week (MIAT) + 2 sessions per week of 30–35 min RT (2–3 sets of 8–12 reps of 8–10 exercises) (RT) **Total duration:** 16 weeks		
Hiensch et al., (2021) [41] (*OptiTrain Breast Cancer Trial*)	**EG1**	**Type of exercise:** Supervised RT-HIIT **Intensity:** 70–80% 1RM (progression when more than 12 repetitions could be performed) (RT) + RPE 16–18 (HIIT) **Volume:** 3 sessions per week; 60 min for each session: 2–3 sets of 8–12 repetitions of 8 exercises (RT) + 3 × 3 min bouts (HIIT) **Total duration:** 16 weeks	Circulating blood cell concentrations (neutrophils and lymphocytes) (*clinical laboratory analysis*) IFN-γ (*Merck Cytomag custom-made*)	No significant differences in IFN-γ
	**EG2**	**Type of exercise:** Supervised AT-HIIT **Intensity:** RPE 13–15 + RPE 16–18 (HIIT) **Volume:** 3 sessions per week; 60 min for each session: 20 min AT + 3 × 3 min bouts (HIIT) **Total duration:** 16 weeks		
	**CG**	**Type of intervention:** Usual care (information about physical activity) **Total duration:** 16 weeks		
Lee et al., (2022) [42]	**EG**	**Type of exercise:** RT **Intensity:** 40–80% 1RM (weekly progression) **Volume:** 2–3 sessions per week; 50 min for each session: 10 min warm-up + 3 sets of 16 repetitions (week 1); 4 sets of 12 repetitions (week 2); and 4 sets of 8 repetitions (weeks 3–12) of 8 exercises + 10 min cool down **Total duration:** 12 weeks	NKCA (*IFN-γ ELISA after activation*)	↑ NKCA in the EG ↑ NKCA in the EG vs. CG
	**CG**	**Type of intervention:** Usual care (activities of daily living) **Total duration:** 12 weeks		
Brown et al., (2023) [43]	**EG**	**Type of exercise:** Supervised (weeks 1–6 + 1 session per month in the following weeks) and unsupervised (>6 weeks, except 1 session per month) RT + unsupervised MIAT **Intensity:** 10RM (RT) + moderate (MIAT) **Volume:** 2 sessions per week; 2−3 sets with a weight that allowed 10 repetitions with correct physical form + 3–6 sessions per week to reach 180 min/week (AT) **Total duration:** 52 weeks	Lymphocyte telomere length (*qPCR*)	No significant differences in lymphocyte telomere length
	**CG**	**Type of intervention:** Instruction to refer to their physician to check which exercise or diet could be safe **Total duration:** 52 weeks		
Arana Echarri et al., (2023) [44]	**EG1**	**Type of exercise:** Partly supervised AT **Intensity:** 55–70% VO_2max_ **Volume:** 2 supervised sessions per week + 1 unsupervised session per week; 35–50 min for each session **Total duration:** 8 weeks	T cells (CD4^+^, CD8^+^, naïve, memory subsets, activated, and TSCM) (*flow cytometry*) B cells (total, naïve, memory, plasmablasts/plasma, and immature) (*flow cytometry*) NK cells (total, effector CD16^+^, and regulatory CD16^−^) (*flow cytometry*) IFN-γ (*ELISpot assay*) IgG antibodies (*ELISA*)	↓ CD4^+^/CD8^+^ ratio in the EG1 vs. EG2 ↑ CD16^−^ regulatory NK cells in the EG1 vs. EG2 No significant differences between groups in T cells, B cells, NK cells, and IFN-γ production
	**EG2**	**Type of exercise:** Remotely supervised AT **Intensity:** 55–70% VO_2max_ **Volume:** 105–150 min each week (minimum bout length of 10 min) **Total duration:** 8 weeks		
Kjeldsted et al., (2025) [45]	**EG**	**Type of exercise:** Supervised HIIT + RT **Intensity:** ≥85% HRmax or RPE ≥ 16 (HIIT) + 65% 1RM increase in load of 10% from week 4 onward if the participant could complete ≥ 17 repetitions (RT) **Volume:** 2–3 sessions per week: 5 min warm-up + 4 × 2 min bouts (HIIT) + 5 min cool down + 3 sets of 12–15 repetitions of 3 exercises (RT) **Total duration:** 18 to 24 weeks	TILs (*manual percentage and digital density*)	↑ Stromal TIL percentage in the CG vs. EG (*manual assessment*) No significant differences between groups in TIL density per 10,000 μm^2^ (*digital analysis*)
	**CG**	**Type of intervention:** Usual care (maintain regular routine) **Total duration:** 18 to 24 weeks		
Ubink et al., (2025) [46]	**EG**	**Type of exercise:** Supervised RT + AT + physical activity recommendations **Intensity:** 70–80% 1RM (gradual progression) (RT) + 50–80% Wmax (AT) + RPE 12–14 (recommendations) **Volume:** 2 sessions per week; 2 sets of 8–12 repetitions (RT); + 30 min (AT); + 3 days per week of 30 min (recommendations) **Total duration:** 6 weeks	T cells (CD3^+^, CD4^+^, CD8^+^, naïve, central memory, effector memory, TEMRA, and regulatory T cells) (*flow cytometry*) B cells (CD19^+^, naïve, memory, plasmablasts/plasma, and immature) (*flow cytometry*) NK cells (CD56^+^, CD56dim/CD16^+^, and CD56bright/CD16^−/+^) (*flow cytometry*) TILs (CD4^+^, CD8^+^, and CD56^+^) and CD4/CD8 ratio (*immunohistochemistry of tumor biopsies with manual and digital quantification*)	↓ CD8^+^ naïve T cells in EG vs. CG ↑ CD107a MFI in NK cells in EG vs. CG ↓ CD4^+^/CD8^+^ ratio in TILs in EG vs. CG ↓ CD4^+^ and CD4^+^ naïve cells T cells, B cells, and CD56dim NK cells in both groups ↑ T cells, CD4^+^ central memory cells, CD4^+^ effector memory cells, CD8+ T cells, and NKT cells in both groups ↓ CD8+ TEMRA cells in the CG No significant differences between groups in other T-cell subsets, B cells, NK cells, and TILs CD56^+^
	**CG**	**Type of intervention:** Usual care **Total duration:** 6 weeks		
Yijing et al., (2025) [47]	**EG1**	**Type of exercise:** Unsupervised LIRT (weights adjusted progressively) + AT **Intensity:** 40–70% 1RM (RPE 13–15) (LIRT) + 30–70% of cardiac reserve (RPE 12–13) (AT) **Volume:** 2–3 sessions per week; 2–3 sets of 15–20 repetitions of 7 exercises (LIRT) + 15–25 to 35–40 min (AT) **Total duration:** 12 weeks (T1) + 12 week follow-up (T2)	NKCA (percentage of CD3^−^CD16/56^+^ perforin^+^, granzyme B^+^, and perforin^+^/granzyme B^+^) (*flow cytometry*)	↑ Perforin^+^ in EG2 vs. EG1 and CG (T1 and T2) ↑ Granzyme B^+^ in EG2 vs. EG1 and CG (T1 and T2) ↑ Perforin^+^/Granzyme B^+^ in EG2 vs. EG1 and CG (T1 and T2) ↑ Granzyme B^+^ in EG1 vs. CG (T2)
	**EG2**	**Type of exercise:** Unsupervised MIRT (weights adjusted progressively) + AT **Intensity:** 50–80% 1RM (MIRT) + 30–70% of cardiac reserve (RPE 12–13) (AT) **Volume:** 2–3 sessions per week; 2–3 sets of 8–12 repetitions of 7 exercises (MIRT); + 15–25 to 35–40 min (AT) **Total duration:** 12 weeks (T1) + 12 week follow-up (T2)		
	**CG**	**Type of intervention:** Health education (diet, physical activity, and psychological regulation) and routine care **Total duration:** 12 weeks (T1) + 12 week follow-up (T2)		

Abbreviations. 1RM, one-repetition maximum; AT, aerobic training; CBC, complete blood count; CG, control group; EG, experimental group; EG1, experimental group 1; EG2, experimental group 2; ELISA, enzyme-linked immunosorbent assay; FOXP3^+^, Forkhead box P3; HIAT, high-intensity aerobic training; HIIT, high-intensity interval training; IFN-γ, interferon-gamma; Igs, immunoglobulins; IgA, immunoglobulin A; IgG, immunoglobulin G; LIRT, low-intensity resistance training; MFI, mean fluorescence intensity; MIAT, moderate-intensity aerobic training; MIRT, moderate-intensity resistance training; NK, natural killer; NKCA, natural killer cytotoxic activity; NKT, natural killer T; NLR, neutrophil-to-lymphocyte ratio; PHA, phytohemagglutinin; qPCR, quantitative polymerase chain reaction; RPE, rate of perceived exertion; RPM, revolutions per minute; RT, resistance training; TILs, tumor-infiltrating lymphocytes; TSCM, T memory stem cell; VO_2peak_, peak oxygen uptake; Wmax, maximum watts. ↑, statistically significant increase after the intervention; ↓ statistically significant decrease after the intervention.

## Data Availability

Data will be provided upon reasonable request to the corresponding author.

## Supplementary Materials

The following supporting information can be downloaded at: https://www.mdpi.com/article/10.3390/cancers18040621/s1, Supplementary Material S1. PRISMA 2020 Checklist; Supplementary Material S2. List of included and excluded studies; Supplementary Material S3. Risk of bias assessment results; Supplementary Material S4. Summary of meta-analysis results including overall and sensitivity analysis.

## Abbreviations

The following abbreviations are used in this manuscript:

1RM	One-repetition maximum
AT	Aerobic training
BC	Breast cancer
CI	Confidence interval
HR_max_	Maximum heart rate
IFN-γ	Interferon-gamma
IL	Interleukin
Ig	Immunoglobulin
MD	Mean difference
NK	Natural killer
NKCA	Natural killer cell activity
PRISMA	Preferred Reporting Items for Systematic Reviews and Meta-Analyses
RCT	Randomized controlled trial
RPE	Rate of perceived exertion
RT	Resistance training
SD	Standard deviation
SMD	Standardized mean difference
TILs	Tumor-infiltrating lymphocytes
TME	Tumor microenvironment
VO_2max_	Maximum oxygen consumption
VO_2peak_	Peak oxygen consumption
