# Supplementary material for: Is There an Immune Effect of Exercise in Patients with Breast Cancer? A Systematic Review and Meta-Analysis
Source: Cancers (Basel). 2026 Feb 13;18(4):621. doi: 10.3390/cancers18040621 (PMC12938651; doi:10.3390/cancers18040621)
Supplement: Supplementary file 1 [file cancers-18-00621-s001.zip › Supplementary material S3.pdf]

	D1	D2	D3	D4	D5	Overall
Nieman et al., (1995)	!	-	-	!	!	-
Fairey et al., (2005)	+	+	+	+	+	+
Hagstrom et al., (2016)	!	!	+	+	+	!
Hagstrom et al., (2018)	+	!	+	+	!	!
Sagarra et al., (2018)	!	!	+	!	!	!
Schmidt et al., (2018)	!	+	+	+	+	!
Ligibel et al., (2019)	+	!	+	+	!	!
Ashem et al., (2020)	!	+	+	+	!	!
Mijwel et al., (2020)	+	+	!	!	+	!
Toohey et al., (2020)	+	!	+	!	+	!
Pagola et al., (2020)	+	+	+	+	!	!
Hiensch et al., (2021)	!	!	+	+	+	!
Lee et al., (2022)	!	!	+	!	!	!
Brown et al., (2023)	!	!	+	+	!	+
Echarri et al., (2023)	!	!	+	!	!	!
Kjeldsted et al., (2025)	+	!	+	+	+	!
Ubink et al., (2025)	+	!	+	+	!	!
Yijing et al., (2025)	!	!	+	+	!	!

+	Low risk
!	Some concerns
-	High risk

D1	Randomisation process
D2	Deviations from the intended interventions
D3	Missing outcome data
D4	Measurement of the outcome
D5	Selection of the reported result
