# Supplementary material for: Is There an Immune Effect of Exercise in Patients with Breast Cancer? A Systematic Review and Meta-Analysis
Source: Cancers (Basel). 2026 Feb 13;18(4):621. doi: 10.3390/cancers18040621 (PMC12938651; doi:10.3390/cancers18040621)
Supplement: Supplementary file 1 [file cancers-18-00621-s001.zip › Supplementary material S4.pdf]

Supplementary material S4. Summary of meta-analysis results including overall and sensitivity analysis

Outcome	Sensitivity analysis	Sample size	SMD (95%CI)	p-value	Heterogeneity
NK	Overall	148 participants<br>EG = 85; CG = 63	-0.24 (-0.57 to 0.09)	*0.15*	$I^2 = 0\%$<br>*p = 0.74*
	Long-term exercise (≥8 weeks)	132 participants<br>EG = 78; CG = 54	-0.27 (-0.62 to 0.08)	*0.13*	$I^2 = 0\%$<br>*p = 0.64*
	Combined exercise	45 participants<br>EG = 23; CG = 22	0.09 (-0.50 to 0.68)	*0.77*	$I^2 = 0\%$<br>*p = 0.92*
NKCA	Overall	111 participants<br>EG = 53; CG = 58	0.13 (-0.64 to 0.90)	*0.74*	$I^2 = 69\%$<br>*p = 0.02*
	Cytotoxic activity	81 participants<br>EG = 38; CG = 43	-0.08 (-1.30 to 1.13)	*0.89*	$I^2 = 79\%$<br>*p = 0.008*
CD3+	Overall	112 participants<br>EG = 64; CG = 48	-0.15 (-0.54 to 0.23)	*0.43*	$I^2 = 0\%$<br>*p = 0.47*
	Long-term exercise (≥8 weeks)	96 participants<br>EG = 57; CG = 39	-0.21 (-0.64 to 0.22)	*0.33*	$I^2 = 5\%$<br>*p = 0.37*
	Combined exercise	45 participants<br>EG = 23; CG = 22	-0.41 (-1.09 to 0.28)	*0.25*	$I^2 = 21\%$<br>*p = 0.28*
CD4+	Overall	100 participants<br>EG = 58; CG = 42	-0.13 (-0.53 to 0.27)	*0.52*	$I^2 = 0\%$<br>*p = 0.95*
CD8+	Overall	100 participants<br>EG = 58; CG = 42	-0.11 (-0.51 to 0.29)	*0.60*	$I^2 = 0\%$<br>*p = 0.94*
B-cells	Overall	100 participants<br>EG = 58; CG = 42	-0.05 (-0.45 to 0.35)	*0.81*	$I^2 = 0\%$<br>*p = 0.89*

Abbreviations. CG, control group; CI, confidence interval; EG, experimental group; SMD, standardized mean difference
